# Upcoming Christmas jump in LIBOR

**DOI:** 10.12688/f1000research.26024.1

**Published:** 2020-10-09

**Authors:** Vikenty Mikheev, Serge E. Miheev

**Affiliations:** 1 Mathematics, Kansas State University, Manhattan, KS, 66502, USA; 2 Applied Mathematics and Control Processes, St. Petersburg State University, St. Petersburg, 198504, Russian Federation

**Keywords:** LIBOR, short term approximation, pattern, swap market, Christmas jump, linear regression

## Abstract

**Background: **London Interbank Offered Rate (LIBOR) exists since 1986 as a benchmark interest rate.

**Methods:** Using two-layer linear regression method, we found a pattern of shortterm nature in LIBOR behaviour.

**Results:** To wit, 2-month LIBOR experiences a jump after Xmas for the last two decades. The direction and size of the jump depend on the data trend on 21 days before Xmas.

**Conclusions:** The obtained results can be used to build a winning strategy on the Swap Market.

## Introduction

In 1986, a new benchmark interest rate was introduced, named the London Interbank Offered Rate (LIBOR). At LIBOR, major banks of the world lend to one another in the international interbank market for short-term loans. From a mathematical point of view, LIBOR is a sequence of daily changing real values. LIBOR data is in open access and can be found on multiple web-sites, for example, here ^1^.

**Figure 1.**
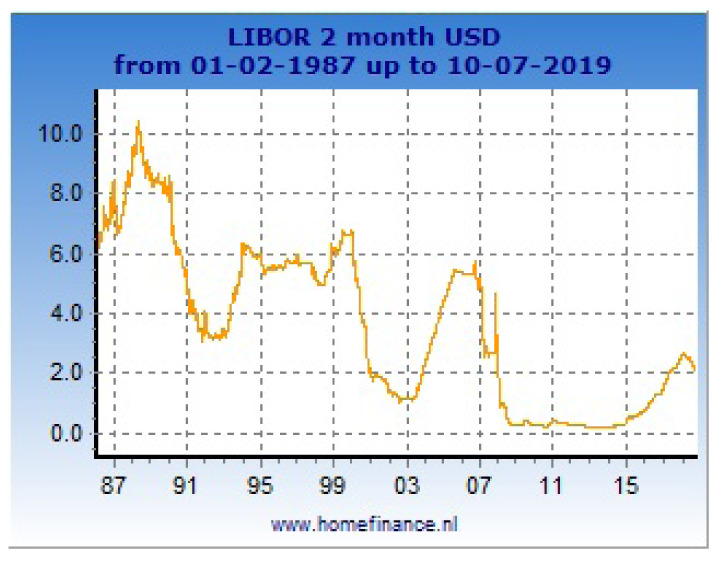
Data of 2-month LIBOR from 1987 to 2019.

**Figure 2.**
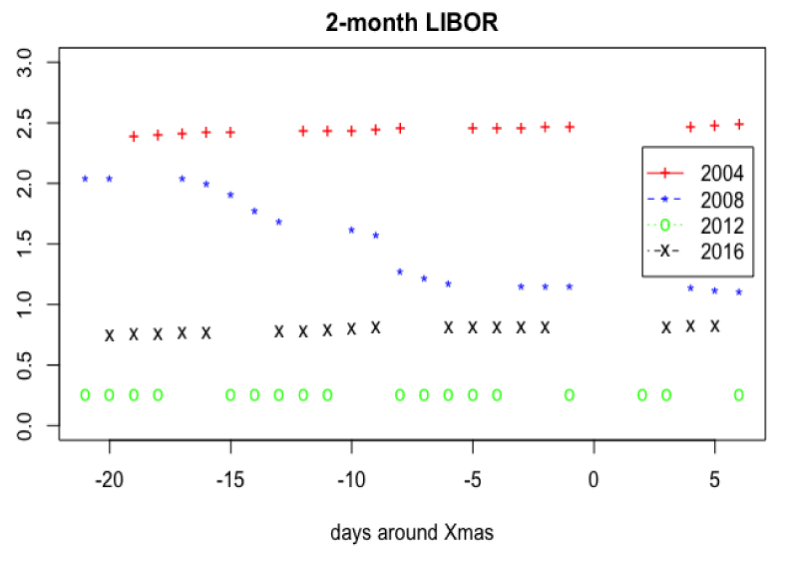
2-month LIBOR values 21 days before Xmas and 6 days after from 2004–2019 years combined.

In Figure 1, one can see a large scale sample of 2-month LIBOR for loans in USD. In Figure 2, the values of 2-month LIBOR 21 days before Xmas and 6 days after from 2004–2019 years were put together.

LIBOR has a crucial role in the Swap Market, where people exchange their loan interests and can win or lose money depending on their right or wrong predictions of LIBOR dynamics. For example, person P got a one-million-dollar loan with 5% interest and person E borrowed the same amount but with the interest 2%+LIBOR. After some time, they decide to exchange their interest rates because P thinks that LIBOR will go lower than 3% but E believes that it will go higher than 3%. Both their opinions are based on some prediction methods, even if it is just an intuition. We intend to bring another prediction tool into the game. A curious reader may find more complex models and measures on LIBOR for different problems ^2– 5^.

**Figure 3.**
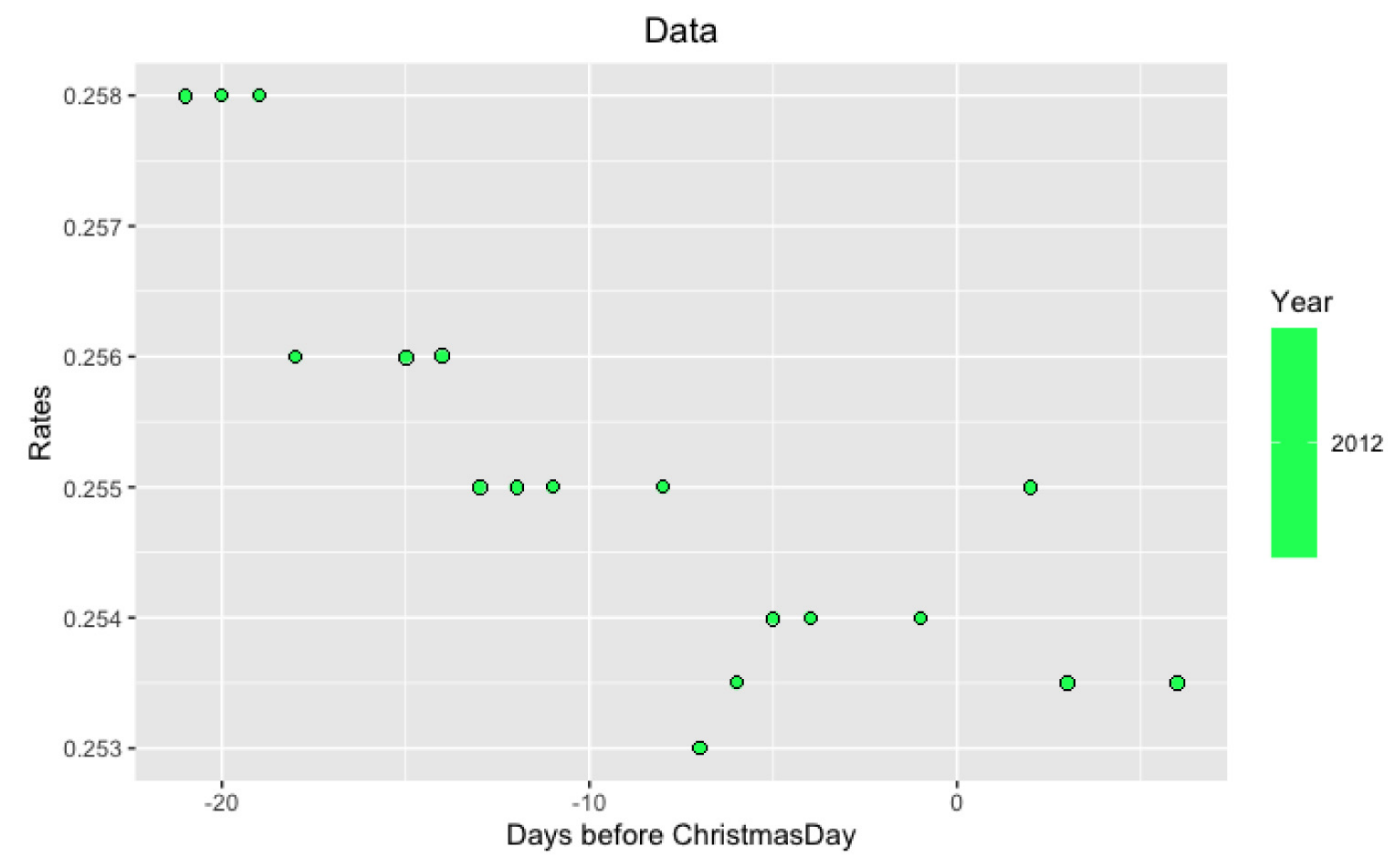
Data from 2012 year around Xmas.

**Figure 4.**
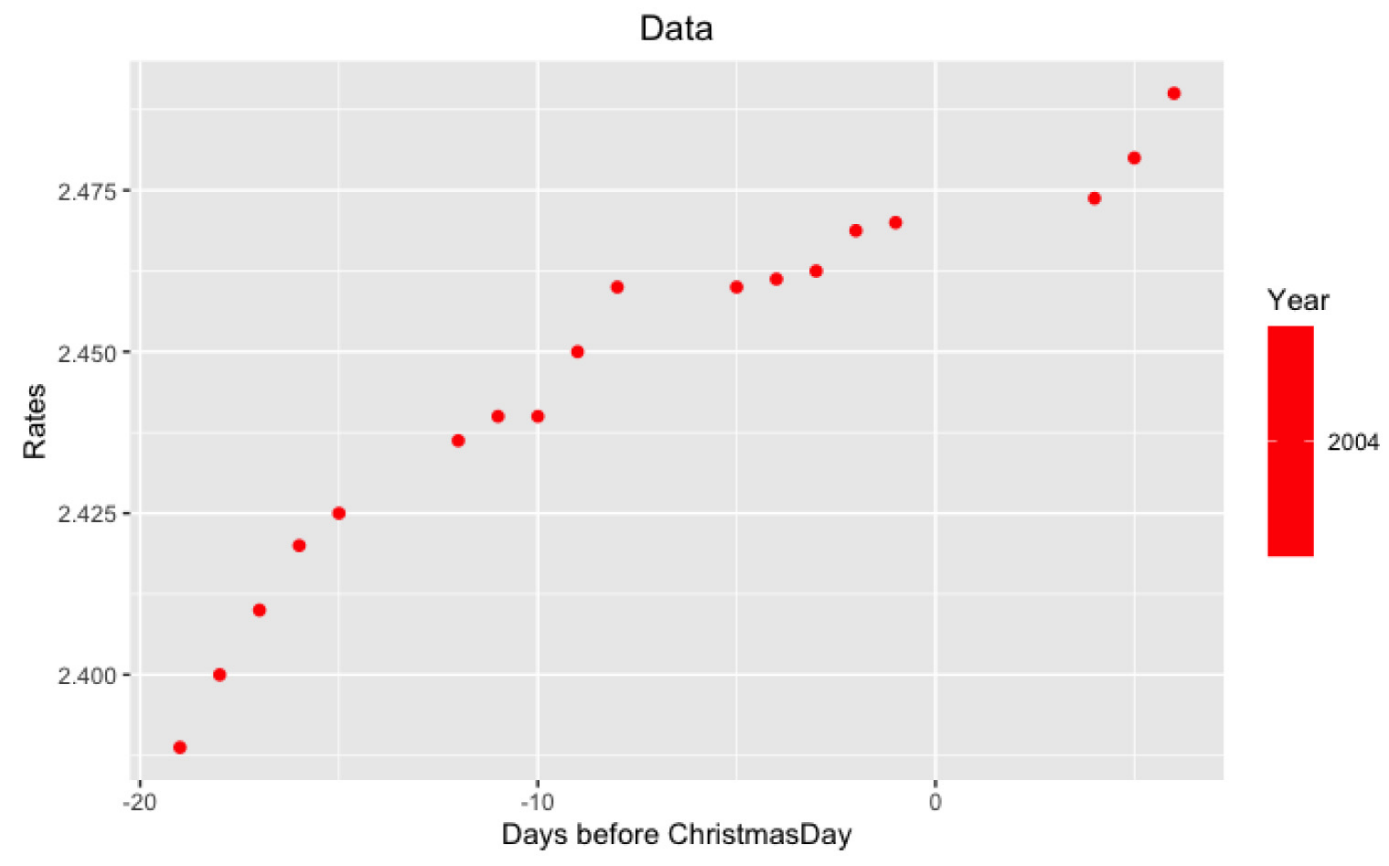
Data from 2004 year around Xmas.

Thus, here we are not interested to LIBOR nature *per se* but in its volatility. More precisely, we study the behavior of LIBOR after Christmas from December 26 to December 31. Examples of such data are in Figure 3 and Figure 4.

Although LIBOR itself is going to disappear in 2021 ^6, 7^, and one can apply our results only for Xmas 2020, we think that the model we introduce here might be useful for short-term analysis in other problems.

So, how does Christmas affect dynamics of LIBOR until the next holiday?

## Methods

### Definition and models

The research we conducted indicates convincingly that a *jump* does exist. But what is a jump in a discrete sequence of numbers? The following seems to be the most acceptable:

**Definition 1.** There are a real number $\bar{x}$ and a real discrete function given tabularly

$$z=\left(\begin{array}{ccc} x_{1} & \cdots & x_{n}\\ y_{1} & \cdots & y_{n} \end{array}\right)\;,$$

where *x*_1_ < *x*_2_ < ... < *x*_*n*_ and $x_{k\;}<\bar{x}<x_{k+1}$ for some natural *k* ∈ [2, *n* − 2]. One chooses approximant *A* : *R* ^*r*^ × *R* ^1^ → *R* ^1^ among functions having continuous derivative by second argument notated as ${A^{\prime }}_{2}$ and chooses some quality criterion *Q*( *z*, *A*( *p*, ·) to minimize by parameter vector *p* ∈ *R* ^*r*^. Then we appoint

$$z_{0}=\left(\begin{array}{ccc} x_{1} & \cdots & x_{k}\\ y_{1} & \cdots & y_{k} \end{array}\right)\;,\;z_{1}=\left(\begin{array}{ccc} x_{k+1} & \cdots & x_{n}\\ y_{k+1} & \cdots & y_{n} \end{array}\right)\;$$

and consider the approximation problem with the criterion *Q*( *z*_0_, *A*( *p*,·)) → min _*p*∈ *R* ^*r*^_. Let its solution be denoted as $\bar{A}(z_{0},x).$ Then we consider the next problem

$$Q(z_{1},A(p,\cdot ))\to \begin{array}{c} \min\\ p\text{∈}R^{r}\;\wedge \;A_{2}^{\prime }(p,\bar{x})={\bar{A}}_{2}^{\prime }(z_{0},\bar{x}) \end{array}\;.$$

Let its solution be denoted as $\bar{A}(z_{1},x).$ Then the difference $\bar{A}(z_{0},\bar{x})-\bar{A}(z_{1},\bar{x})$ is the jump at $\bar{x}$ .

In other words, the jump at $\bar{x}$ of a discrete function given by a tabular is the difference at $\bar{x}$ between the obtained solutions of two approximation problems of the same type, the first problem is formulated on the left part of the table, the second problem is formulated on the right part of the table and must keep at $\bar{x}$ trend (i.e. derivative) of the first problem solution. The left part corresponds to the nodes less than $\bar{x}$ , the right part corresponds to the nodes greater than $\bar{x}$ .

In our case, $\bar{x}$ =Dec 25).

It is easy to see that the so-defined jump depends on the type of approximation and on the amount of the input data. On top of that, we have to decide the amount of input data in *z*_0_. Notice that the amount of the data in *z*_1_ is only three pairs (date, LIBOR of this date) because there are exactly three working banking days between Xmas and New Year’s Eve (NYE). The data source is available in 1 or in many other sources.

Variability of the data due to random factors leads to the choice of the simplest approximation. We use linear approximating functions, which coefficients may be found by linear regression with its own quality criterion. We restrict ourselves to LIBOR data for the last 22 years, because it is natural to expect the evolution of LIBOR behavior over the years.

So, for year *j* in set *J* taken sequentially with no gaps from {1997, ..., 2019} data are taken for 15 banking days $x^{\prime }$ _−15_,..., $x^{\prime }$ _−1_ (corresponding to 21 calendar days) preceding Xmas of year *j*. Since all the days are in December, we may refer to them just by number without problem of passing days to another month: $x^{\prime }$ _−15_,..., $x^{\prime }$ _−1_ ⊂ {4,...,24}. Moreover, for simplicity of following constructions we may decrease them by 25, i.e. xi:=x′i−25 , *i* = −15,...,−1. Therefore, *x*_−15_,..., *x*_−1_ ⊂ {−21,...,−1}. Each *x _i_* corresponds to *y _i_*, which is the annual interest rate of LIBOR for 2 months on day *x*_*i*_. Using them we build a linear regression

$$\hat{y}(x)=a_{j}x+b_{j},\;(1)$$

or $y_{i}=\hat{y}(x)+\varepsilon_{i},$ where *x* is a December day minus 25 and *ε*_*i*_ is the error.

**Figure 5.**
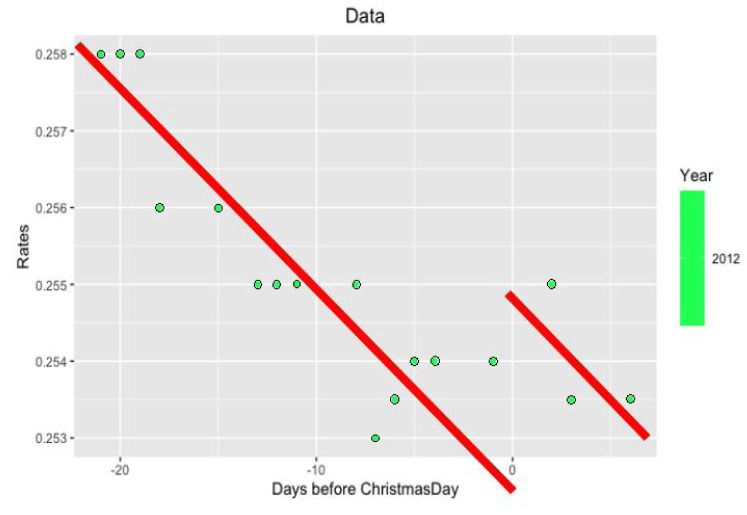
Approximation on data of 2012 year.

**Figure 6.**
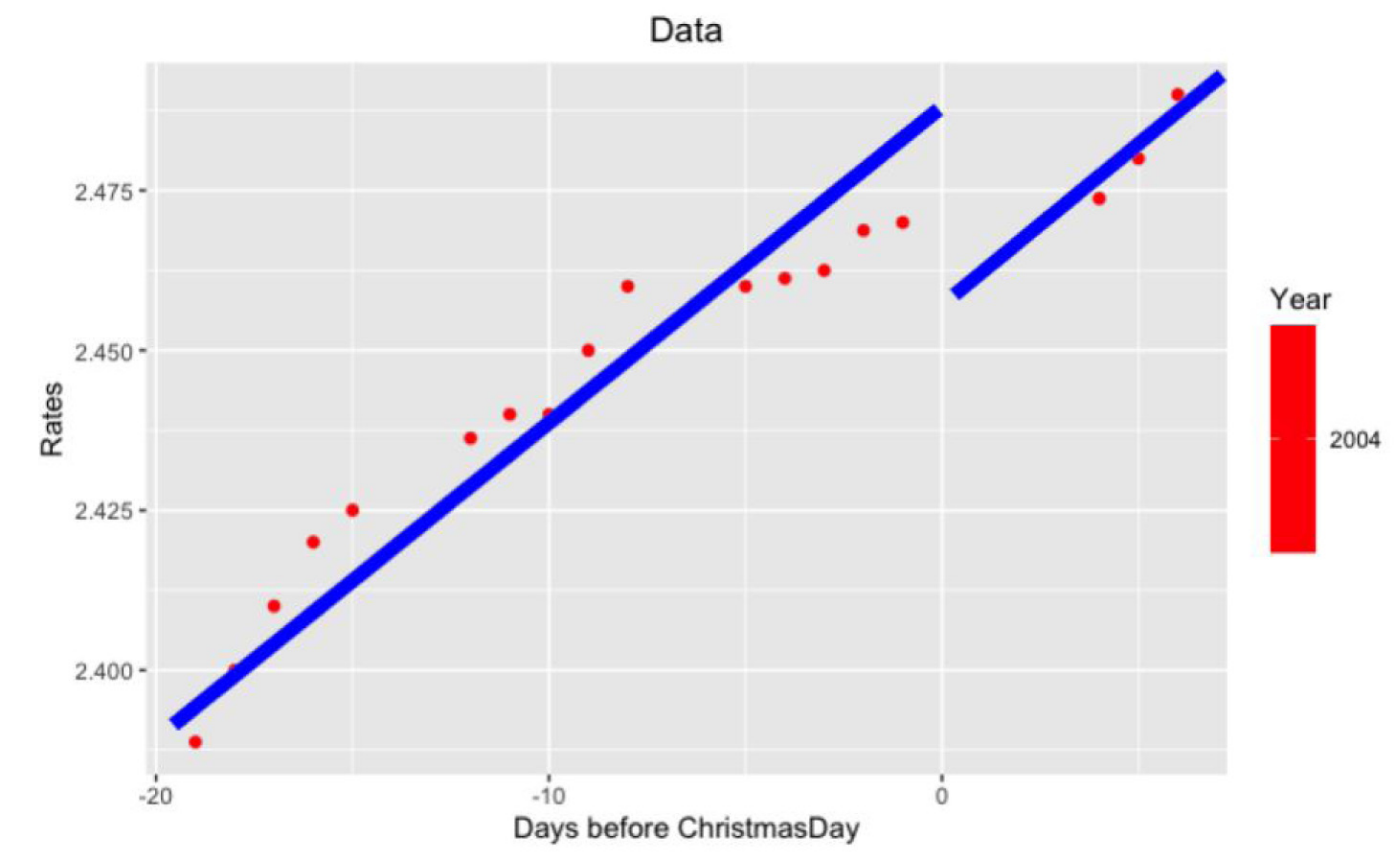
Approximation on data of 2004 year.

That is, in terms of Definition 1, $\bar{A}(z_{0},x)=a_{j}(z_{0})x+b_{j}(z_{0}).$ Since the current trend of LIBOR (meaning the rate of growth or decrease) does not change a lot over a short time interval, it is almost the same before and after Xmas. Therefore, we seek an approximation after Xmas in the following form: $\hat{y}(x)={a^{\prime }}_{j}x+{b^{\prime }}_{j},$ where *x* is a December day decreased by 25 and ${a^{\prime }}_{j}=a_{j}=\bar{A}(z_{0},\bar{x})\text{.}$ Hence, the second approximation has only one unknown parameter, ${b^{\prime }}_{j}\text{.}$ It can also be found by linear regression or can be calculated simply as the average of values *y _i_* − *a _j_* *x _i_*, where *i* runs 1,2,3 and *x _i_* ∈ {27 − 25,...,31 − 25} = {2,...,6} (There are exactly three bank days between Xmas and New Year.) The examples of such approximations are seen in Figure 5 and Figure 6.

Notice that on the last Figure 5 and Figure 6 with data for 2012 and 2004 years and their regressions, the y-axes have different scales.

Thus, for each selected year *j* there is a relationship $(b_{j},a_{j})\to {\hat{b}}_{j}.$ According to Definition 1 the difference $\Delta_{j}\;\text{:=}\;{\hat{b}}_{j}-b_{j}$ is the jump we have been looking for. Having such connections over 23 years, one can try to find a pattern. To do that, we turn to linear-quadratic regression on two-dimensional nodes. This time the approximating function has the form:

$$F(a,b):\text{=}\;\beta_{0}+\beta_{1}a+\beta_{2}b+\beta_{3}ab\;(2)$$

with an approximation table *F*_*J*_ ( *a*_*j*_, *b*_*j*_) ≈ *Δ*_*j*_, *j* ∈ *J* ⊂ {1995,...,2019}. Sub index *J* at *F* points at which subset of years over the past 23 has been chosen to construct the regression. The remaining years will be used to verify the statistical reliability of the result.

### Why 15 week days before and 3 week days after Xmas?

On one hand, we want as much data for our approximation as possible. On the other hand, the longer the time interval, the less accurate the trend on the end of the interval. Someone could say: ”Why don’t you take a more complex approximant to capture more complicated futures of the time series?” Well, that would require even more data for statistical power of such approximant. Since we want to detect a short-term pattern, we should avoid such approach. It made sense to take a number of days before Xmas divisible by 5, so each week day would appear evenly. After trying 25, 20, 15, 10, 5, we found the model worked best with 15 days.

Regarding 3 LIBOR days after Xmas, the same logic explained above is applicable here too. Again, empirically we have found that 3 days work the best. Notice every year has exactly 3 LIBOR days between Xmas and NYE. It is possible that NYE plays a big role in that.

## Results

We conducted the process above for several different numbers of years for *F* regression (from 5 to 20 years), different LIBOR data (overnight, 1 month, 2 months, etc.). The most convincing results have been obtained with the following setups: 21 calendar day regression for each year from 15-year intervals; 2-month-loan values of LIBOR.

Observe the results in the Table 1.

**Table 1.** Model *Δ* ≈ β _0_ + β _1_ *a* + β _2_ *b* + β _3_ *ab* and its predictions with p-values *p* for 2019 year.

Data	2000–14	2001–15	2002–16	2003–17	2004 – 18	2005 – 20
Pred. on	2015	2016	2017	2018	2019	2020
$\hat{\beta_{0}}$ $\hat{\beta_{1}}$ $\hat{\beta_{2}}$ $\hat{\beta_{3}}$	-2.96E-3 -9.286 1.8E-4 1.91521	4.1E-4 -9.337 -0.002209 1.99021	4.1E-4 -9.321 -0.00220 1.98676	0.00327 -9.278 -0.00204 2.02619	0.00473 -9.265 -0.00238 2.016	0.00477 ( *p*=0.157) -9.26577 ( *p*=1.93e-12) -0.002346 ( *p*=0.113) 2.01876 ( *p*=2.90e-08)
Adj *R* ^2^	0.98	0.99	0.99	0.99	0.99	0.99
RSS	0.01178	0.00844	0.00844	0.00984	0.00815	0.00854
Pred. val Real val	-0.0709 -0.0599	-0.0306 -0.0269	-0.0548 -0.0291	-0.0180 -0.0228	-0.0085 -0.0188	wait for data till 12/24/2019
${\hat{L}}^{mean}$ *L* ^*mean*^	0.5024 0.5134	0.8146 0.8183	1.5967 1.6224	2.6222 2.6174	1.8509 1.8404	wait for data till 12/24/2020
Error	-0.0110	-0.0037	-0.0257	0.0048	0.0105	

So, our prediction for the jump formula after Xmas 2020 are:

$${\hat{\Delta }}_{2020}=0.004770-9.265766a_{2020}-0.002346b_{2020}+2.018758a_{2020}b_{2020}\;(3)$$

The 95%-confidence intervals for the coefficients *β*_0_, *β*_1_, *β*_2_, *β*_3_ in ( 3) are (-0.00217, 0.01171), (-9.77318, -8.75835), (-0.00536, 0.00066) and (1.72466, 2.31286), respectively.

It may be activated at Dec 24 2020 as following:

At this day extract data { *y*_*i*_} from 1 for bank days $\left\{{x^{\prime }}_{i}\right\}$ since Dec 21 till Dec 24 (in 2020, of course). Build 15 pairs ( *x*_*i*_, *y*_*i*_), *i* = −15,...,−1, where xi=x′i−25 . Put them into any program to find linear regression, for example, into our code in R, which is available as *Extended data* ^8^. The result of the regression is two numbers: that corresponding to free term is *b*_2020_, the other is *a*_2020_. Substitution of them to ( 3) yields the jump.

The prediction of the jump can be used to predict the mean LIBOR after Xmas and before NYE ( $L_{j}^{mean}).$ Let us show some formulas.

According to Definition 1 the jump with approximations above is $\Delta_{j}={\hat{b}}_{j}-b_{j}$ , where ${\hat{b}}_{j}$ = arg min _*b*_ $\{\sum_{i=1}^{3}{\left(a_{j}x_{i}+b-y_{i}\right)}^{2}\},$ which is equivalent to ${\hat{b}}_{j}=\frac{1}{3}\sum_{i=1}^{3}(y_{i}-a_{j}x_{i}).$ Hence

$$\Delta_{j}=\frac{1}{3}\sum_{i=1}^{3}(y_{i}-a_{j}x_{i})-b_{j}=\frac{1}{3}\sum_{1}^{3}y_{i}-\frac{1}{3}\sum_{i=1}^{3}\left(a_{j}x_{i}+b_{j}\right).\;(4)$$

Notice that the last term in ( 4) is just a predicted mean value of LIBOR between Xmas and NYE $({\hat{L}}_{j}^{mean}),$ according to the regression for *j*-th year. Thus,

$$L_{j}^{mean}={\hat{L}}_{j}^{mean}+\Delta_{j}={\hat{L}}_{j}^{mean}+{\hat{\Delta }}_{j}+(\Delta_{j}-{\hat{\Delta }}_{j}).$$

If as estimate of $L_{j}^{mean}$ we take ${\hat{L}}_{j}^{mean}+{\hat{\Delta }}_{j},$ then its absolute error is equal to ${\hat{\Delta }}_{j}-\Delta_{j}.$

The latter difference according to our calculations for years 2015, 2016, 2017, 2018, 2019 was always less by absolute value than $|{\hat{\Delta }}_{j}|.$

## Conclusion

We have found a short-term pattern in LIBOR dynamics. Namely, the 2-month LIBOR experiences a jump after Xmas. The sign and size of the jump depends on data trend on 21 days before Xmas. The results are obtained in the form of the jump *per se* and as mean predicted value of LIBOR between Xmas and NYE. A swap market player may try to use this information to predict behaviour of LIBOR to do a better game on his part. For Xmas of 2020, on a date of Dec 24, one can compute *a* and *b* according to ( 1) on 21 calendar days and use the formula ( 3) to predict the jump after Xmas.

In the pre-print ^9^ of this paper, one can find our predictions for the jump after Xmas of 2019 and see that later data from the event confirmed it.

## Data availability

### Source data

All data used in this paper can be found at IBORate ( http://iborate.com/usd-libor/) ^1^. 

### Extended data

**The code used to develop the model is available at:** https://github.com/keshmish/Chistmas-Jump-in-LIBOR/. 

**Archived code at time of publication:** https://doi.org/10.5281/zenodo.3977133 ^8^.

**License:** MIT License.

## References

[1] IBORate: LIBOR database. Cited 15 Jan 2020. Reference Source

[2] Jamshidian F : LIBOR and swap market models and measures. *Financ Stoch.* 1997;1:293–330. 10.1007/s007800050026

[3] Schoenmakers J : Robust Libor Modelling and Pricing of Derivative Products. Chapman and Hall/CRC, New York.2005. Reference Source

[4] Moreni N Pallavicini A : Parsimonious HJM modelling for multiple yield-curve dynamics. *Quant Finance.* Routledge,2014;14(2):199–210. 10.1080/14697688.2013.829242

[5] Hinch M McCord M McGreal S : LIBOR and interest rate spread: sensitivities of the Australian housing market. *Pac Rim Prop Res J.* Routledge,2019;25(1):73–99. 10.1080/14445921.2019.1610594

[6] Heltman J : Libor is going dark in 2021, and some banks aren’t ready. American Banker.2018. Cited 15 Jan 2020. Reference Source

[7] Farrar D : You might have heard that LIBOR is going away.... *Consumer Financial Protection Bureau*.2019. Cited 15 Jan 2020. Reference Source

[8] Mikheev V : Code in R for Xmass Jump in Libor. 10.5281/zenodo.3977133

[9] Mikheev V Miheev SE : Christmas Jump in LIBOR. *Arxiv.* 2019. Reference Source 10.12688/f1000research.26024.2 PMC10403742 37547625

