# Supplementary material for: Repeating Christmas jump in LIBOR
Source: F1000Res. 2023 Jul 27;9:1221. Originally published 2020 Oct 9. [Version 2] doi: 10.12688/f1000research.26024.2 (PMC10403742; doi:10.12688/f1000research.26024.2)
Supplement: Peer review history

# Peer review: Reviewer response for version 2

**DOI:** 10.5256/f1000research.152396.r191301

**Published:** 2023-08-04

**Authors:** FAUZIA MUBARIK

**Affiliations:** 1 Department of Management Science, National University of Modern Languages, Islamabad, Pakistan

No further comments to make.

Is the work clearly and accurately presented and does it cite the current literature?

Yes

If applicable, is the statistical analysis and its interpretation appropriate?

Partly

Are all the source data underlying the results available to ensure full reproducibility?

Yes

Is the study design appropriate and is the work technically sound?

Yes

Are the conclusions drawn adequately supported by the results?

Yes

Are sufficient details of methods and analysis provided to allow replication by others?

Yes

Reviewer Expertise:

Financial Economics, Islamic Finance

I confirm that I have read this submission and believe that I have an appropriate level of expertise to confirm that it is of an acceptable scientific standard.

# Peer review: Reviewer response for version 1

**DOI:** 10.5256/f1000research.28719.r127765

**Published:** 2022-05-30

**Authors:** FAUZIA MUBARIK

**Affiliations:** 1 Department of Management Science, National University of Modern Languages, Islamabad, Pakistan

The article has a good readability and qualifies to be approved with reservations. This article has an interesting way forward to identify and recognize the most recent developments in the Alternative Reference Rates (ARR); Secured Overnight Financing Rate (SOFR) of United States, Sterling Overnight Index Average (SONIA) of England, Euro Short-Term Rate (ESTR) of European Union, Swiss Average Rate Overnight (SARON) of Switzerland and Tokyo Overnight Average Rate (TONAR) of Japan introduced in the global financial market as the successors to LIBOR.

 The article can be published after incorporating the following suggestions/recommendations;

1. The in-text citations are missing.
2. The results can be strengthened through empirical support. 2021 and 2020 were big years for LIBOR research, please cite some of this research – or conduct more of your own – to strengthen your results. See this Congressional Report, this from KKR Investments, and this from the Bank for International Settlements, for example.
3. The researcher may explain whether any windows are created to study the jumps.
4. Is it a two step study, first of 21 days and 6 days; second of 15 days and 3 days?
5. Is the prediction in the jump studied through mean of the regression method?

Is the work clearly and accurately presented and does it cite the current literature?

Yes

If applicable, is the statistical analysis and its interpretation appropriate?

Partly

Are all the source data underlying the results available to ensure full reproducibility?

Yes

Is the study design appropriate and is the work technically sound?

Yes

Are the conclusions drawn adequately supported by the results?

Yes

Are sufficient details of methods and analysis provided to allow replication by others?

Yes

Reviewer Expertise:

Financial Economics, Islamic Finance

I confirm that I have read this submission and believe that I have an appropriate level of expertise to confirm that it is of an acceptable scientific standard, however I have significant reservations, as outlined above.

# Response

**Published:** 2023-02-15

**Authors:** Vikenty Mikheev

**Affiliations:** Kansas State University, USA

1. If you could specify where exactly those missing citations were supposed to be, it would help us a lot.

 2. The articles you mentioned are talking about LIBOR transition, which is a little unrelated to our topic. Unfortunately, adding data after 2020 is hard since the LIBOR got mostly shut down in 2021. There above, you mentioned similar to LIBOR data where the proposed here method could be tried on. This is a very useful hint. Thanks!

 3. We hope that the section "Why 15 week days before and 3 week days after Xmas?" explains this very reasonable question.

 4,5. "Two-step" in our research stays for two-step regression. First, we find (by a regression) the parameters of a function for each year from 1997 to 2019. Second, we do another regression, this time on those found parameters to predict the next year.

# Peer review: Reviewer response for version 1

**DOI:** 10.5256/f1000research.28719.r123668

**Published:** 2022-03-02

**Authors:** Victor Malyutin

**Affiliations:** 1 Institute of Mathematics of the National Academy of Sciences of Belarus, Minsk, Belarus

The behavior of LIBOR after Christmas from December 26 to December 31 is studied in the paper. The Authors use linear regression to find the regression coefficients for data before Xmas and jump after Xmas for each selected year. To get the jump prediction formula the linear-quadratic regression on two-dimensional nodes is used. The prediction of the jump can be used to predict the mean LIBOR after Xmas and before NYE. Presented results demonstrate that predicted results approximate real values well.

Is the work clearly and accurately presented and does it cite the current literature?

Yes

If applicable, is the statistical analysis and its interpretation appropriate?

Yes

Are all the source data underlying the results available to ensure full reproducibility?

Yes

Is the study design appropriate and is the work technically sound?

Yes

Are the conclusions drawn adequately supported by the results?

Yes

Are sufficient details of methods and analysis provided to allow replication by others?

Yes

Reviewer Expertise:

Mathematical statistics

I confirm that I have read this submission and believe that I have an appropriate level of expertise to confirm that it is of an acceptable scientific standard.
